# Premature Birth is an Independent Risk Factor for Early Adiposity Rebound: Longitudinal Analysis of BMI Data from Birth to 7 Years

**DOI:** 10.3390/nu12123654

**Published:** 2020-11-27

**Authors:** Maria Elisabetta Baldassarre, Antonio Di Mauro, Margherita Caroli, Federico Schettini, Valentina Rizzo, Raffaella Panza, Alessia De Giorgi, Manuela Capozza, Margherita Fanelli, Nicola Laforgia

**Affiliations:** 1 Department of Biomedical Science and Human Oncology, Neonatology and Neonatal Intensive Care Unit, “Aldo Moro” University of Bari, 70100 Bari, Italy; dimauroantonio@msn.com (A.D.M.); federico.schettini@uniba.it (F.S.); rizzovale.vr@gmail.com (V.R.); raffaella.panza@uniba.it (R.P.); alessiadegiorgi90@gmail.com (A.D.G.); manuelacapozza26@gmail.com (M.C.); nicola.laforgia@uniba.it (N.L.); 2 Independent Researcher, 72021 Francavilla Fontana, Italy; margheritacaroli53@gmail.com; 3 Department of Interdisciplinary Medicine, “Aldo Moro” University of Bari, 70100 Bari, Italy; margherita.fanelli@uniba.it

**Keywords:** adiposity rebound, infant, premature, pediatric obesity, complementary feeding, weaning, body mass index

## Abstract

Adiposity rebound (AR) refers to the second rise of the body mass index (BMI) curve that usually occurs physiologically between five and seven years of age. AR timing has a great impact on patients’ health, since early adiposity rebound (EAR) is associated with the development of metabolic syndrome later in life. We aimed to investigate the prevalence of EAR in a cohort of inborn preterm infants admitted to the Neonatal Intensive Care Section of the Policlinico University Hospital of Bari, Italy. Secondarily, we assessed whether some determinants such as (1) gender; (2) delivery mode; (3) birth weight and classification into small, normal, or large for gestational age; (4) type of feeding; (5) breastfeeding duration; (6) timing of introduction of solid food; (7) parental education; and (8) parental pre-pregnancy body mass index (BMI) influenced EAR in this cohort. The tertiary aim was to evaluate the prevalence of obesity or being overweight at seven years of age in children according to early versus timely AR. This is a prospective, population-based longitudinal study conducted at the Neonatal Intensive Care Section of the Policlinico University Hospital of Bari, Italy. Inborn preterm infants admitted to the neonatal ward between 2009 and 2011 were eligible. Enrolled preterm infants were evaluated at birth and at 1, 3, 6, 9, 12, 15, 18, and 24 months and 3, 4, 5, 6, and 7 years of age. Weight and height data were analyzed, and BMI was calculated. AR was assessed in the growth trajectory in a body mass index (BMI) plot. Of the 250 preterm newborns included, 100 completed the seven-year follow-up and entered the final analysis, 138 were lost during the seven-year follow-up, and in 12 cases parents withdrew over the course of the study. The prevalence of EAR in our cohort of preterm newborns was 54% at seven years of age. Early adiposity rebound was associated with being large for gestational age (LGA) at birth. No other factors were associated with EAR. Early adiposity rebounders had a significantly higher BMI at seven years compared to children with timely AR (17.2 ± 2.7 vs. 15.6 ± 2.05, *p* = 0.021). No significant differences were found in the prevalence of obesity or being overweight at seven years of age in children with early or timely AR (29% vs. 14%, *p* = 0.202). Ex-preterm infants have an increased risk of EAR. Since EAR may lead to long-term detrimental health effects with the onset of various chronic diseases (e.g., obesity, metabolic syndrome, etc.), healthcare providers should be prepared to counteract its occurrence, especially in delicate sub-populations of infants.

## 1. Introduction

Obesity in childhood is a major concern for public health worldwide and its prevalence is expected to increase in the near future [1]. To counteract such a worrying trend, efforts should be made to understand early predictors of obesity in order to implement timely preventive interventions in the first stages of life.

Rolland-Cachera et al. identified a typical growth trajectory of the body mass index (BMI) plot. Physiologically, BMI rapidly increases during the first year of life, then it decreases and reaches a nadir between five and seven years of life; thereafter it increases again throughout childhood. The exact point of the BMI curve nadir with the immediate subsequent increase represents the so-called adiposity rebound (AR) [2]. If the BMI curve reaches its low before five years of age, then the diagnosis of early adiposity rebound (EAR) can be made. An EAR can be predictive of adult obesity and other obesity-related comorbidities [3,4,5].

Early adiposity rebound has recently been a matter of research, however its pathophysiology as well as related risk factors are still under debate [6]. Growing evidence supports that early life events are pathogenically linked to non-communicable diseases later in life and some authors speculated that even prenatal features may predispose to obesity and cardiovascular diseases [7]. The greater risk for later obesity and cardiometabolic sequelae in preterm babies seems to be particularly linked to the rapid weight gain from birth to infancy and the early increase of fat mass and BMI [8,9,10].

Furthermore, the first few months after birth may be regarded as a crucial period in terms of later effects on weight gain and childhood obesity [11]. This may be due to the different impact on metabolism of breastfeeding versus formula feeding, and of age at the start of complementary feeding.

Preterm infants are defined by the WHO as “born alive before 37 weeks of pregnancy” and the prevalence of preterm birth ranges from 5% to 18%. According to the programming theories, preterm infants during early stages of life show different postnatal patterns of growth (catch-up growth) than term infants, with a greater likelihood of developing obesity, cardiovascular disease, and diabetes in adulthood.

Due to the significant improvements of neonatal intensive care and the high survival of preterm infants, their lifelong management has become an important field of research with impacts for the primary care provider. Primary care pediatricians have to screen all ex-preterm patients for obesity and should advise behavioral interventions and provide intensive counseling to children at high risk. So far, there are no clear recommendations for ex-preterm infants on the timing of initiation of complementary feeding, and only few data are available regarding the optimal timing of solid food introduction in ex-preterm infants and the effect on being overweight and having obesity later in life [12].

The main aim of this population-based longitudinal study is to evaluate the rate of EAR in preterm newborns. The secondary aim is to investigate the role of parental and neonatal factors and nutritional characteristics in EAR development. The tertiary aim is to calculate rates of obesity and being overweight at seven years of age in infants with and without EAR.

## 2. Methods

### 2.1. Study Design

This study is a prospective, population-based longitudinal study, conducted in the Neonatal Intensive Care Section of the Department of Biomedical Science and Human Oncology of Aldo Moro University in Bari, Italy. All inborn preterm infants between 2009 and 2011 were eligible for the study. Inclusion criteria were (a) Italian language-speaking parents and (b) gestational age (GA) at birth between 23 and 36 weeks. Exclusion criteria included genetic syndromes, congenital and/or malformation disorders; any kind of surgery; major neurological, immune, metabolic, cardiac, or renal diseases; monozygotic twins; and absence of parental consent.

All consecutive newborns were recruited over the first week of life, during hospitalization. Clinical visits were scheduled at 3, 6, 9, 12, 15, 18, and 24 months of corrected age and at 3, 4, 5, 6, and 7 years of age. Up to 3 years of age, infants’ growth was evaluated according to the adjusted postnatal age (calculated as postnatal age in weeks minus 40 weeks plus gestational age in weeks) [13]; whereas after 3 years, chronological age was considered. Auxological data at birth were collected from hospital charts. Throughout the follow-up, weight was measured on an electronic scale, and length was the average of two valid measurements. The measurements were always made in the same place and with the same precision instruments. We excluded from the final analysis (a) children who developed spasticity problems with difficult anthropometric assessment and (b) children with special healthcare needs. The study was approved by the Ethics Committee of the Policlinico Hospital in Bari (study nr. 4122, 20/2/2013). The parents signed an informed consent before including their infants in the study.

### 2.2. Outcomes Assessment

The primary outcome was the prevalence of EAR in a cohort of Italian preterm newborns.

BMI was calculated based on the anthropometrical data collected over the 7-year follow-up. The timing of AR was defined as the age at the lowest BMI [4]. An AR occurring before the fifth year of life was considered EAR [2].

The secondary outcomes aimed to evaluate any associations between EAR and neonatal/infant factors, such as (1) gender (male/female); (2) type of delivery (caesarean/vaginal); (3) birth weight and classification into small, normal, or large for gestational age; (4) type of feeding (breast milk/formula/mixed); (5) duration of breastfeeding; (6) timing of introduction of solid food; (7) parental education; and (8) parental BMI. Such variables were selected for their potential role in increasing the risk for adiposity rebound or obesity in adult age, or both.

All newborns were classified according to birth weight corrected for gestational age for Italian newborns in normal for gestational age (NGA, weight between 10th and 90th percentile), small for gestational age (SGA, weight <10th percentile), and large for gestational age (LGA, weight >90th percentile) [14].

The timing of the introduction of solid foods was regarded as the corrected age (in months) at the earliest administration of any solid food: before 4 months (corresponding to 16 weeks), between 4 and 6 months (corresponding to 24 weeks), and after 6 months.

Breastfeeding and its duration were categorized as (a) no breastfeeding, (b) less than 6 months, (c) from 6 to 12 months, and (d) more than 12 months.

Parental and social determinants were also investigated. Parental education level was defined as high (any college/associate degree, bachelor, or postgraduate degree) or low.

Parental BMI calculation was based on self-reported height and pre-pregnancy weight, and was categorized as underweight (BMI < 18.5 kg/m^2^), normal weight (BMI 18.5–24.9 kg/m^2^), overweight (BMI 25.0–29.9 kg/m^2^), or obese (BMI ≥ 30.0 kg/m^2^).

Children’s BMI at 7 years of age was categorized as: normal (<85th percentile), overweight (≥85th, <97th percentile), or obese (≥97th percentile) according to the WHO BMI-for-age sex-specific growth charts [15].

### 2.3. Statistical Analysis

A simple description of data was made in terms of mean, standard deviation, and percentage depending on the type of data.

The association between EAR and (1) sex, (2) type of delivery, (3) birth weight classification, (4) type of feeding at 6 months of age (breast milk/formula/mixed), (5) duration of breastfeeding, (6) timing of introduction of solid food, (7) parental education, and (8) parental BMI was assessed by contingency tables and χ^2^ test. Subsequently, a multivariate logistic regression model was created. Gestational age at birth, duration of breastfeeding, time at the first food introduction, and BMI were compared between children with or without EAR by Student’s unpaired *t*-test. For all tests, a *p*-value of <0.05 was considered statistically significant. SPSS v23 (IBM, Armonk, NY, USA) was employed to analyze the data.

## 3. Results

**Figure 1:**
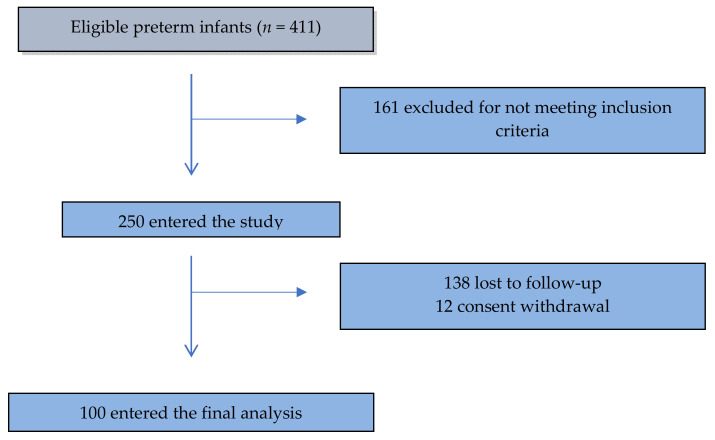
STROBE flow-chart.

Out of 411 eligible preterm infants, 161 were excluded after not meeting inclusion criteria; 250 children entered the study but 138 were lost during the seven-year follow-up and in 12 cases parents withdrew over the course of the study. Most of these newborns were lost at one year of life, hence the data were insufficient to carry out a statistical analysis. One hundred (40%) completed the seven-year follow-up and entered the final analysis (Figure 1).

Demographical and nutritional characteristics of the sample according to timing of adiposity rebound are described in Table 1 and Table 2. Parental characteristics are shown in Table 3.

AR age (years) is shown in Table 4. Overall, EAR was found in 54 (54%) of preterm infants.

No significant differences between children with or without EAR were observed for gestational age at birth (*p* = 0.85), duration of breastfeeding (*p* = 0.49), or timing of first solid food introduction (*p* = 0.98).

EAR was not significantly associated with gender (*p* = 0.75), type of delivery (*p* = 0.40), birth weight (*p* = 0.09), type of feeding (*p* = 0.48), duration of breastfeeding (*p* = 0.83), timing of introduction of solid food (*p* = 0.97), maternal education (*p* = 0.27) and BMI (*p* = 0.98), or paternal education (*p* = 0.28) and BMI (*p* = 0.18). The results of the multivariate analysis for risk factors associated with EAR are shown in Table 5.

At the seven-year follow-up visit, 6% of children were overweight and 23% obese. Ex-preterm infants with EAR have a significantly higher BMI at seven years compared to those with normal AR (17.2 ± 2.7 vs. 15.6 ± 2.05, *t* = 2.385, *p* = 0.021). Furthermore, the prevalence of obesity in subjects with EAR was higher, although not statistically significant (29% vs. 14%, *p* = 0.20).

## 4. Discussion

Our study shows that 54% of ex-preterm children had an early adiposity rebound with BMI rebound at or before the fourth year of age. This is a significantly higher prevalence in comparison to other European studies on children born at term, in which around 30% of children had EAR [2,16]. This high prevalence of EAR is significantly related to higher BMI at seven years of age and may possibly increase the risk of obesity and other non-communicable diseases in adulthood [17], as previously stated in a recent systematic review and meta-analysis in which childhood obesity was found to be a predictor of morbidity in adulthood [18].

In our cohort, none of the investigated neonatal factors were associated with EAR, gender included, albeit female obesity susceptibility is a well-known phenomenon [19]. It is possible that obesity development or excessive weight gain later in life are more related to pubertal hormonal changes than EAR.

Some authors have investigated whether cesarean section (CS) is associated with excessive weight and obesity in children, with contrasting results [20,21,22]. Babies born by cesarean section are not exposed to maternal microbiota, so they have lower diversity and richness of the gut microbiome compared to babies delivered vaginally [23]. This might influence both the composition and metabolic functions of gut microbiota and their effects on the storage of dietary nutrients, predisposing infants to being overweight or having obesity [24,25,26]. We have not found any association between cesarean section and EAR.

SGA neonates are more prone to early adiposity rebound, obesity, and metabolic syndrome, especially those featuring the early catch-up growth phenotype [27]. In our cohort, SGA neonates did not show EAR, but this finding could have been affected by the small sample size of the cohort.

Conversely, LGA newborns had increased odds of EAR compared to their NGA counterparts, confirming that LGA status is a risk factor for being overweight or obese in early childhood, as demonstrated by previous studies [28,29].

In our sample of preterm infants, parental BMI and educational status did not show a significant relationship with EAR, in contrast with other studies [16,30].

To date, breastfeeding is widely advocated as a protective factor against childhood obesity. Breastfed infants feature an ideal growth model in which growth occurs regularly without rapid weight gain. Recent literature has widely shown that infants who are exclusively breastfed are less prone to having excess weight during late infancy, in part because breastfeeding in comparison to formula milk determines lower insulin response, which prevents excessive fat deposition and decreases adiposity, thus helping prevent chronic diseases later in adulthood. Breast milk also features a high amount of bifidobacteria, which have been proven scarce in the gut of overweight or obese children. Lastly, children who were breastfed in the early stages of life show healthier food habits afterwards, preferring fruit and vegetables as compared to their formula-fed counterparts [31].

The switch from exclusively milk-based nutrition to solid foods represents a delicate moment for the infant. This timepoint was defined as “weaning” until some years ago, but today the term “complementary feeding” is preferred as it underlines the role of nutritional integration to breast milk. The age of four to six months is regarded as the most appropriate for the introduction of complementary feeding, since after six months of life breast milk is not sufficient to meet the growing child’s needs, with special regard to proteins, iron, zinc, and vitamins [32].

Over the last two decades several studies have assessed the impact of timing of complementary feeding [33,34,35,36,37,38,39,40,41,42] and/or breastfeeding [41,43] on adiposity rebound and obesity in childhood, but the results are still rather conflicting (Table 6).

Our study failed to demonstrate any different prevalence of EAR according to type of breastfeeding, formula or mixed. Our results show only a weak association between breastfeeding and normal adiposity rebound, possibly as a consequence of the limited number of exclusively breastfed infants in our cohort (15%).

Furthermore, there are few data regarding the effect of the timing of complementary feeding on the risk of being overweight or obese in ex-preterms. A recent study showed that weaning of these infants is characterized by great heterogeneity [44,45]. In our cohort, neither early (<4 months of corrected age) nor late (>6 months of corrected age) weaning time showed to be related to EAR. Recently, an observational cohort study concluded that preterm infants with complementary feeding at ≤26 weeks of corrected age featured a higher BMI at one year of age compared those who started complementary feeding after 26 weeks of corrected age [46].

However, a recent systematic review assessing the relationship between the timing of complementary feeding in preterm infants and the incidence of being overweight failed to draw clear conclusions due to the shortage of large randomized controlled trials [47].

We are aware of some limitations of our study. First, our inclusion criteria could represent a bias since preterm infants with significant comorbidities have been excluded. Secondly, our follow-up stopped at seven years, possibly missing or underestimating any association of EAR with obesity or being overweight that occurred after seven years of age [48]. Furthermore, the timing of adiposity rebound is influenced by many other confounding factors, such as maternal smoking during pregnancy, socio-economic status of the family, screening time, diet, and early protein intake during neonatal admission [6,49,50].

This paper is the first to show EAR prevalence in a cohort of ex-preterm newborns and to evaluate the role of different neonatal factors on EAR with a follow-up up to seven years.

We demonstrated a high prevalence of EAR in ex-preterm newborns that is not influenced by several neonatal factors, confirming that prematurity per se represents a high-risk condition. Our data suggest that pediatricians should carefully follow ex-preterm children to screen for excessive weight or obesity very early because of their confirmed predisposition [51].

Further studies are needed to confirm our data in ex-preterm babies, focusing on the role of other factors such as feeding type and quality of solid foods during weaning, with the aim of developing new preventive strategies. A case-control study on ex-preterm infants matched with their term counterparts would confirm the role of prematurity per se on EAR.

## 5. Conclusions

The timing of adiposity rebound has an impact on adverse health outcomes. More than 50% of preterm newborns in our cohort showed early adiposity rebound, revealing that prematurity per se represents a high-risk condition. Determinants such as gender, type of delivery, birth weight, feeding type and breastfeeding duration, timing of introduction of solid food, parental education, and parental BMI are not associated with EAR. Since EAR may lead to long-term detrimental health effects with the onset of various chronic diseases (e.g., obesity, metabolic syndrome, etc.), healthcare providers should be prepared to counteract its occurrence, especially in delicate sub-populations of infants.

## Figures and Tables

**Table 1:** Neonatal characteristics according to timing of adiposity rebound.

Neonatal Demographics	EAR	NO EAR	Total	Test	*p*-Values
Male, *n* (%)	24 (44%)	19 (41%)	43 (43%)	χ^2^ = 0.10	*p* = 0.75
Female, *n* (%)	30 (56%)	27 (59%)	57 (57%)		
Vaginal birth, *n* (%)	9 (17%)	5 (11%)	14 (14%)	χ^2^ = 0.69	*p* = 0.40
Caesarean section, *n* (%)	45 (83%)	41 (89%)	86 (86%)		
Small for Gestational Age, *n* (%)	7 (15%)	8 (15%)	15 (15%)	χ^2^ = 4.80	*p* = 0.09
Adequate for Gestational Age, *n* (%)	37 (80%)	36 (67%)	73 (73%)		
Large for Gestational Age, *n* (%)	2 (4%)	19 (18%)	12 (12%)		
ELBW, *n* (%)	1 (2%)	0 (0%)	1 (1%)	χ^2^ = 2.94	*p* = 0.40
VLBW, *n* (%)	0 (0%)	2 (4%)	2 (2%)		
LBW, *n* (%)	5 (11%)	5 (9%)	10 (10%)		
NBW, *n* (%)	40 (87%)	47 (87%)	87 (87%)		
Gestational age at birth, weeks mean (SD)	34 (2)	34 (2)	34 (2)	*t* = 0.19	*p* = 0.85

EAR—Early Adiposity Rebound; ELBW—Extremely Low Birth Weight; VLBW—Very Low Birth Weight; LBW—Low Birth Weight; NBW—Normal Birth Weight.

**Table 2:** Nutritional characteristics according to timing of adiposity rebound.

Nutritional Characteristics	EAR	NO EAR	Total	Test	*p*-Values
Type of feeding at 6 months of age					
Exclusive breast milk, *n* (%)	7 (13%)	8 (17%)	15 (15%)	χ^2^ = 1.46	*p* = 0.48
Exclusive formula, *n* (%)	17 (31%)	18 (39%)	35 (35%)		
Mixed, *n* (%)	30 (56%)	20 (44%)	50 (50%)		
Duration of breastfeeding, mean (SD)	6 (9)	5 (4)	6 (7)	*t* = 0.7	*p* = 0.49
Duration of breastfeeding, distribution					
No breastfeeding, *n* (%)	17 (32%)	18 (39%)	35 (35%)	χ^2^ = 0.88	*p* = 0.83
≤6 months, *n* (%)	26 (48%)	19 (41%)	45 (45%)		
6–12 months, *n* (%)	5 (9%)	5 (11%)	10 (10%)		
>12 months, *n* (%)	6 (11%)	4 (9%)	10 (10%)		
Solid foods introduction					
<4 months of corrected age, *n* (%)	1 (2%)	1 (2%)	2 (2%)	χ^2^ = 0.05	*p* = 0.98
4-6 months of corrected age, *n* (%)	44 (81%)	38 (83%)	82 (82%)		
>6 months of corrected age, *n* (%)	9 (17%)	7 (15%)	16 (16%)		

EAR—Early Adiposity Rebound.

**Table 3:** Parental characteristics according to timing of adiposity rebound.

Parental Characteristics	EAR	NO EAR	Total	Test	*p*-Values
**Mother educational level**					
low, *n* (%)	19 (36%)	12 (26%)	31 (32%)	χ^2^ = 1.23	*p* = 0.27
high, *n* (%)	33 (63%)	34 (74%)	67 (68%)		
**Father educational level**					
low, *n* (%)	22 (41%)	14 (30%)	36 (36%)	χ^2^ = 1.14	*p* = 0.28
high, *n* (%)	32 (59%)	32 (70%)	64 (64%)		
**Mother BMI**					
BMI <18.5 kg/m^2^, *n* (%)	0	0	0	χ^2^ = 0.04	*p* = 0.98
BMI 18.5–24.9 kg/ m^2^, *n* (%)	40 (74%)	34 (74%)	74 (74%)		
BMI 25.0–29.9 kg/ m^2^, *n* (%)	10 (18%)	9 (20%)	19 (19%)		
BMI ≥30.0 kg/ m^2^, *n* (%)	4 (7%)	3 (6%)	7 (7%)		
**Father BMI**					
BMI < 8.5 kg/ m^2^, *n* (%)	0	0	0	χ^2^ = 3.46	*p* = 0.18
BMI 18.5–24.9 kg/ m^2^, *n* (%)	22 (41%)	11 (24%)	33 (33%)		
BMI 25.0–29.9 kg/ m^2^, *n* (%)	27 (51%)	30 (65%)	57 (58%)		
BMI ≥30.0 kg/ m^2^, *n* (%)	4 (7%)	5 (11%)	9 (9%)		

BMI—body mass index, EAR—Early Adiposity Rebound.

**Table 4:** Adiposity rebound distribution per years.

AR (year)	Total *n* (%)
2	15 (15%)
3	25 (25%)
4	14 (14%)
5	17 (17%)
6	21 (21%)
7	8 (8%)

AR—adiposity rebound.

**Table 5:** Multivariate analysis for risk factors associated with early adiposity rebound.

Variables	Early AR	
	OR	*p*-Value
Male vs. female	0.82	0.70
Vaginal birth vs. caesarean section	0.38	0.20
**Birth Status**		
SGA vs. NGA	0.85	0.80
LGA vs. NGA	6.63	0.04
**Feeding**		
Formula feeding vs. Breastfeeding	5.53	0.11
Mixed vs. Breastfeeding	5.20	0.06
Breastfeeding duration (≤6 months vs. >6 months)	0.54	0.4
Solid food introduction * (more than 6 months vs. 4–6 months)	1.40	0.65
Mother educational level (none/basic vs. higher)	1.75	0.44
Father educational level (none/basic vs. higher)	0.96	0.95
**Mother BMI**		
25–29.9 vs. 18.5–24.9 kg/m^2^	2.45	0.20
>30 vs. 18.5–24.9 kg/m^2^	0.89	0.91
**Father BMI**		
25–29.9 vs. 18.5–24.9 kg/m^2^	0.56	0.28
>30 vs. 18.5–24.9 kg/m^2^	0.70	0.70

* The ≤4 months category was not considered in the model due to the small number of cases. BMI—body mass index; SGA—small for gestational age; LGA—large for gestational age; NGA—normal for gestational age; AR—adiposity rebound; OR—adjusted odds ratio.

**Table 6:** Summary of recent studies assessing feeding factors associated with adiposity rebound in childhood.

Reference	Population	Sample Size (*n*)	Factors Associated with Adiposity Rebound
Ariza et al. [33], 2004	Cross sectional study on 5–6-year-old Hispanic children	250	Age of introduction of solid food not associated with being overweight
Burdette et al. [34], 2006	Prospective cohort study on healthy 5-year-old full-term European or Afro-American infants	313	Breastfeeding and timing of the introduction of complementary foods not associated with adiposity at age 5 y
Brophy et al. [35], 2009	Sub-cohort study on 7–9-year-old singleton children	17,561	Early introduction of solid food (<3 months) associated with major risk of obesity
Butte [36], 2009	Cross-sectional study on children of families with one overweight 4–19-year-old child	1030	Age of introduction of solid food not a risk factor for obesity
Neutzling et al. [37], 2009	Birth cohort study on 14–16-year-old boys	1204	No difference in the proportion of overweight or obese children between earlier (<4 months) or later (>4 months) introduction of solid food
Seach et al. [38], 2010	Cohort study on Australian children at risk of atopy	307	Delayed introduction of solid food associated with reduced odds of child being overweight or obese
Gooze et al. [39], 2011	Longitudinal birth cohort study of randomly sampled births in 10-year-old American children	6750	Major prevalence of obesity in earlier introduction of solid food (<3 months)
Caleyachetty et al. [40], 2013	Prospective cohort study on healthy Indian children with pregnancy-related maternal risk factors	484	No association between age of introduction of solid food and sum of skinfold thicknesses
Turčić Škledar et al. [41], 2015	Prospective cohort study on healthy Caucasian 6–7-year-old children	302	Important role of longer duration of breastfeeding (≥3 months) and later introduction of complementary food (>6 months) in preventing obesity
Moschonis et al. [42], 2017	Prospective cohort studies on European and British 6–7-year-old children	24,952	Early feeding not consistently associated with growth and adiposity indices. No clear influence can be observed.
Wu et al. [43], 2020	Longitudinal study on children with high genetic risk of obesity	5266	Reduced BMI growth during childhood in case of exclusive breastfeeding to 5 months

## Abbreviations

AR	adiposity rebound
BMI	body mass index
EAR	early adiposity rebound
GA	gestational age
LGA	large for gestational age
NCDs	non-communicable diseases
NGA	normal for gestational age
SGA	small for gestational age
